# Pyrodextrin enhances intestinal function through changing the intestinal microbiota composition and metabolism in early weaned piglets

**DOI:** 10.1007/s00253-020-10419-z

**Published:** 2020-03-03

**Authors:** Lihui Zhu, Rongrong Liao, Weilong Tu, Yonghong Lu, Xuan Cai

**Affiliations:** 1 Institute of Animal Husbandry & Veterinary Science, Shanghai Academy of Agricultural Sciences, Shanghai, 201106 People’s Republic of China; 2 Shanghai Engineering Research Center of Breeding Pig, Shanghai, 201106 People’s Republic of China

**Keywords:** Microbiota, Piglets, Pyrodextrin, Short-chain fatty acids

## Abstract

Pyrodextrin (PD) is prepared from starch by heat treatment and is resistant to amylase. We hypothesized that PD might have prebiotic potential affecting the microbiota composition, because it contains a non-digestible portion that may behave as dietary fiber. This study investigated the effects of PD supplementation on growth performance, gut morphology, short-chain fatty acids (SCFAs), and the bacterial community in weaned piglets receiving dietary supplementation of 0.5% PD. The piglets in the PD (treated) groups showed greater antioxidant capacity and feed efficiency (*P* < 0.05), as well as improved intestinal morphology in comparison with the piglets in the weaned (control) group. Gut microbiota profiles were assessed through 16S rRNA sequencing on the ileum contents and feces of early weaned piglets. Several genus-level enrichments and depletions were observed in response to PD treatment. Of note, PD supplementation decreased the relative abundance of pathogenic organisms, including *Defluviicoccus* and *Gardnerella*, while markedly increasing that of commensal bacteria (genera *Psychrobacter* and *Prevotella*), which have important roles in nutrient absorption and immune response regulation. The most notable effect in the PD treatment groups was increased production of SCFAs in the feces of PD-treated weaned piglets. Correlation analysis revealed that the improvement in SCFAs was positively correlated with the increase in SCFA-producing bacteria. Overall, this study provides a more comprehensive understanding of the effects of PD supplementation on the fecal microbial community and the modulation of SCFA production in early weaned piglets, thus indicating that PD can be used to alleviate weaning stress in piglets.

**Electronic supplementary material:**

The online version of this article (10.1007/s00253-020-10419-z) contains supplementary material, which is available to authorized users.

## Introduction

Healthy development of piglets provides strong overall benefits in pig production. The stresses caused by changes in the environment and nutrient sources after weaning in piglets are responsible for substantial losses each year in the swine industry in China, thus resulting in reduced growth rates and nutrient absorption efficiency and increased susceptibility to gut dysfunction, diarrhea, and high morbidity and mortality (Aumaitre and Corring 1978).

In addition, weaning-induced decreased intestinal digestive enzyme activities, damaged tight junction proteins, impaired immune response, and increased cytokines were also reported in several papers studying in post-weaning piglets (Boudry et al. 2004; Hu et al. 2013; Pié et al. 2004). Therefore, functional diets or nutrients that alleviate weaning stress in piglets are becoming major areas of swine nutrition research worldwide.

Non-digestible and functional carbohydrates are commonly referred to as dietary fiber, which provide health benefits such as intestinal motility (Watanabe et al. 2018) and intestinal immune functions (Fastinger et al. 2008; Miyazato et al. 2016). Heating starch, occasionally with acid, causes a modification of polymer structures, thus resulting in different characteristics such as solubility in cold water, decreased viscosity, and resistance to alpha-amylase. Starches processed through these methods are referred to as pyrodextrins (PDs). PD is water-soluble and expected to behave like dietary fiber, which exerts various physiological functions, including increasing mineral absorption, preventing diarrhea, and improving intestinal bacteria (Sajilata et al. 2006). Miyazato et al. (2016) found that continuous intake of soluble dietary fiber enhances the intestinal immune response in a dose-dependent manner, resulting in increased total immunoglobulin A (IgA) levels and production of short-chain fatty acids (SCFAs) in the intestinal tract of mice. Furthermore, the soluble fiber resistant maltodextrin is a slowly fermentable prebiotic fiber that affects the gut microbiota, producing a marked change in the *Bifidobacteria* count in humans (Burns et al. 2018). Dietary supplementation with prebiotics can effectively increase the number and proportion of intestinal beneficial bacteria (Gibson 1999), prevent intestinal inflammation, and improve nutrient metabolism (Goetze et al. 2008).

As we previously observed that the addition of PD to feed reduced the incidence of diarrhea among weaning piglets (results shown in the online supplemental methods and materials), we speculate that PD may be used to alleviate weaning stress. To study the influences of PD on post-weaning piglets, here, a specially prepared PD concentration was included at 0.5% in a basal diet (results shown in the online supplemental methods and materials). The bacterial composition in the post-weaning piglets after PD treatment was evaluated by 16S rRNA sequencing and compared with that in the absence of PD. Similarly, we also compared serum biochemical changes and the production of SCFAs. In addition, intestinal morphology analysis was performed to compare the intestinal barrier function in the PD-treated post-weaning piglets with that in the control animals.

## Methods and materials

### Animals and experiment design

This study was approved by the Ethics and Animal Welfare Committee of the Shanghai Academy of Agricultural Sciences, Shanghai, China.

The test material was Tumihappy™ PD prepared (0.5%) from cornstarch (PD; Matsutani Chemical Industry Co., Ltd., Hyogo, Japan) according to our previous study (online supplemental methods and materials). A total of 24 litters of landrace piglets (8–12 piglets per litter) with similar birth weights were divided into weaning (control) and PD-treated groups with 12 litters per group, according to the average weight. The piglets were kept with the sow in conventional farrowing pens and suckled until 21 days of age. From 14 to 42 days of age, piglets in the weaning (control) group were given ad libitum access to the basal diet, and the PD-treated group was fed the basal diet supplemented with 500 mg/kg of PD. At 21 days of age, all the piglets were weaned and moved from the farrowing pens to nursery pens without mixing any litters. The temperature in the farrowing and nursery pens was maintained at approximately 30 °C.

### Sample collection

At 25 and 42 days of age, one piglet from each litter was selected, respectively, thus resulting in a total of 12 piglets per treatment (one per litter) in a single day of age, and blood samples were collected from the anterior vena cava. The selected piglets had body weights close to the average for the litter. Serum was separated by centrifugation at 3500×*g* for 15 min at 4 °C and stored at − 20 °C until analysis for cortisol and antioxidative physiological markers.

At 42 days of age, one piglet from each litter was selected as the body weight close to the average for the litter, anesthetized by intramuscular injection of sumianxin II (0.2 mL/kg body weight), and then euthanized, after which gut samples were collected. The entire small intestine was carefully removed and placed on ice. Pieces of the small intestine, approximately 2 cm in length, were resected from the middle portion of the ileum and fixed in 4% neutral buffered formalin for hematoxylin-eosin (HE) staining. Meanwhile, the contents of the ileum and rectum were also carefully collected. Other segments of the ileum were rinsed thoroughly with physiological saline, frozen in liquid nitrogen, and stored at − 80 °C until further quantitative RT-PCR analyses. In addition, at 25 days of age, feces from the control and the PD-treated groups (one piglet per litter) were collected by placing collection bags at the tails of the piglets.

### Serum biochemical analysis

Serum concentrations of cortisol (CSB-E06811p, Cusabio Inc., Wuhan, China), IgA (CSB-E13234p, Cusabio Inc., Wuhan, China), and tumor necrosis factor α (TNF-α; CSB-E16980p, Cusabio Inc.) were quantified with competitive enzyme-linked immunosorbent assay according to the manufacturer’s protocol. Endotoxin was assayed with the tachypleus amebocyte lysate method (EC80545S, Xiamen Bioendo Tech., Xiamen, China). The activity of superoxide dismutase (SOD) and the content of malondialdehyde (MDA) were determined to evaluate the antioxidant ability and lipid peroxidation in pigs and were measured with assay kits according to the manufacturer’s instructions (Nanjing Jiancheng Bioengineering Institute, Jiangsu, China). The concentrations of albumin, blood glucose, triglyceride (TG), total cholesterol (TC), and blood urea nitrogen (BUN) and the activity of alkaline phosphatase (AKP), aspartate aminotransferase (GOT), and alanine aminotransferase (GPT) were also measured with commercial assay kits from Nanjing Jiancheng Bioengineering Institute.

### HE staining

Samples of the ileum were dehydrated, embedded in paraffin, sectioned (~ 4 μm), and stained with HE. The villus height, crypt depth, and number of goblet cells in the crypts were measured in ten well-oriented villi and crypts by using a light microscope (Olympus, Tokyo, Japan) and linear ocular micrometer (Olympus).

### Quantitative RT-PCR

Total RNA was extracted from ileum tissues with TRIzol reagent (Takara Biotechnology, Dalian, China) according to the manufacturer’s instructions. The quantity and quality of the RNA were measured with a Nanodrop 2000 (NanoDrop Technologies, Wilmington, DE, USA) instrument and verified by electrophoresis on a 1.5% agarose gel. Total RNA (0.5 mg) was reverse-transcribed with random primers according to the manufacturer’s protocol (Takara Biotechnology). The resulting complementary DNA was diluted and used as a PCR template to evaluate gene expression. Quantitative RT-PCR was conducted on an ABI7500 Real-Time Quantitative PCR System (Thermo fisher, Waltham, USA) using SYBR Premix Ex Taq kits (Takara Biotechnology) under the following conditions: pre-denaturation at 95 °C for 30 s and 40 cycles of 95 °C for 5 s, 60 °C for 30 s, and 72 °C for 15 s. A dissociation curve was constructed at the end of the reaction to ensure that only one amplicon was amplified. Primers for the genes of interest were designed on the basis of the pig (*Sus scrofa*) sequence (Supplemental Table S1). All experiments were repeated in triplicate, and β-actin was used as an internal control for normalization. All mRNA relative expression levels were calculated with the comparative Ct method (Livak and Schmittgen 2001).

### 16S rRNA sequencing

Microbial genomic DNA was extracted from the feces and ileum contents by using a QIAamp DNA stool mini kit (Qiagen, Hilden, Germany) according to the manufacturer’s instructions. The concentrations and integrity of genomic DNA were verified with a Nanodrop 2000 spectrophotometer and 1.5% agarose gel electrophoresis. The variable region of 16S rRNA V4 was amplified by using the universal primer sequence, 343F: 5′-TACGGRAGGCAGCAG-3′ and 798R: 5′-AGGGTATCTAATCCT-3′. Library construction was performed on barcoded V4 PCR amplicons and sequenced on the Illumina MiSeq PE250 platform (San Diego, CA, USA). Raw data are now available at NCBI in the Sequence Read Archive database under accession no. SRR8733085-SRR8733156.

### Data processing

Raw sequences were first filtered, and reads with adapter contamination at the ends of the reads, reads < 50 bp, and reads with low quality (quality score < 20) were removed with the Trimmomatic program (Bolger et al. 2014). Subsequently, the qualified double-ended raw data were spliced to obtain paired-end sequences with a maximum overlap of 200 bp using Flash (Magoc and Salzberg 2011). Clean tag sequence was then obtained by using the split libraries software in QIIME (Caporaso et al. 2010b) to remove sequences containing N bases in the paired-end sequences, single base repeat sequences greater than six, and sequences with a length less than 200 bp. Finally, the UCHIME (Edgar et al. 2011) software was used to remove the chimerism in clean tags, and valid tags were obtained for subsequent operational taxonomic unit (OTU) partition. Subsequently, sequence clustering was performed with the Vsearch algorithm (Rognes et al. 2016) and clustered into OTUs. The most abundant sequence in each OTU was selected as a representative.

The taxonomy of each OTU was assigned by blasting the representative sequence against the Greengenes reference database (Release 13.8, http://greengenes.secondgenome.com/) by using the RDP classifier Naive Bayesian classification algorithm (Wang et al. 2007). Unknown archaeal or eukaryotic sequences were filtered and removed. According to sequence alignment, the PyNAST (v0.1) software (Caporaso et al. 2010a) was used to construct the phylogenetic relationship of OTUs of representative sequences, and a phylogenetic tree was obtained. Diversity index data were analyzed statistically with analysis of variance, and significant differences between group means were determined with the least significant difference test.

Functional genes were predicted through PICRUSt according to the abundance of the OTU level, and predicted genes were assigned Cluster of Orthologous Groups of proteins (COG) and Kyoto Encyclopedia of Genes and Genomes (KEGG) pathway annotations; differences among groups were compared with STAMP (http://kiwi.cs.dal.ca/Software/ STAMP) by using two-sided Welch’s *t* test.

### Short-chain fatty acid detection

For SCFA detection, 1 g of each sample (feces or ileum contents) was diluted with distilled water, homogenized, and centrifuged 12,000×*g* for 10 min. Metaphosphoric acid (0.2 mL, 25% w/v) containing crotonic acid solution was added into 1 mL of supernatant. After storage overnight at − 20 °C, the samples were centrifuged for 10 min at 12,000×*g*. The supernatant was filtered through a 0.22-μm filter, and 0.5-μL filtrate was injected into a gas chromatograph (7890B, Agilent Technologies, CA, USA) equipped with a flame ionization detector and a capillary column (30 m × 0.32 mm × 0.25 μm film thickness). To measure SCFAs, we used crotonic acid as an internal standard (1.077 mg/L). The column, injector, and detector temperatures were 130, 180, and 180 °C, respectively. Hydrogen gas, produced by a gas generator (Parker ChromGas, Parker Hannifin Corporation, MN, USA), was used as the carrier gas at a flow rate of 40 mL/min. A standard SCFA mixture containing acetate, propionate, butyrate, isobutyric acid, pentanoic acid, and isopentanoic acid was used for calculation, and the results are expressed in milligrams per gram of sample.

### Statistical analysis

Statistical analyses were performed in SPSS v21.0 (IBM Co., NY, USA). Values are means ± SEM unless otherwise noted. The statistical significance threshold was set at *P* < 0.05, and *P >* 0.05 was considered no statistical significance.

## Results

### Effects of PD supplementation on growth performance and serum parameters in early weaned piglets

**Fig. 1:**
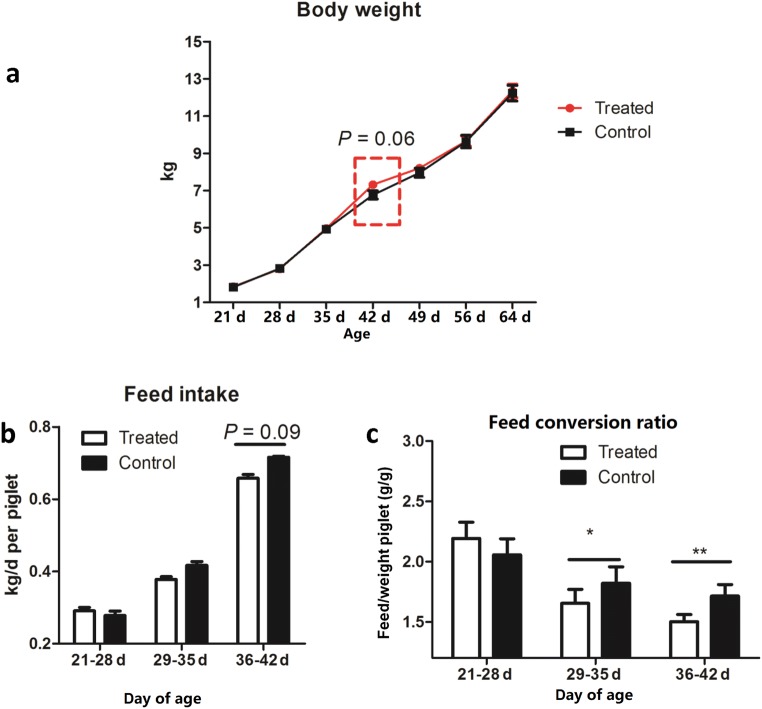
Growth performance and dietary intake of weaned piglets at different days of age: body weight (**a**), feed intake (**b**), and feed conversion ratio (**c**)

No significant differences in growth performance were observed between the two groups (the PD-treated piglets and the control animals), except at 42 days of age, at which the body weight of the PD-treated piglets tended to be higher (*P* = 0.06) than that in the control weaned piglets (Fig. 1a). However, from 36 to 42 days of age, dietary PD supplementation tended to decrease feed intake (*P* = 0.09, Fig. 1b) but significantly decreased the feed conversion ratio (feed conversion ratio (FCR) = feed ingested (g) / weight gain (g)) from 29 to 42 days of age (*P* < 0.05, Fig. 1c), thus indicating that dietary PD increased the feed efficiency. As shown in Table 1, PD supplementation had no effect on serum parameters, but PD significantly increased the concentrations of albumin and TC and the SOD activity of early weaned piglets at 42 days of age. In addition, no significant differences in all measured serum parameters were observed between the PD-treated piglets and the control weaned piglets at 25 days of age. According to biochemical parameters, no negative value was found in the PD-treated piglets compared with that in the control weaned piglets.

**Table 1:** Effects of PD supplementation on serum parameters in early weaned piglets

	25 days				42 days			
	Control		Treated		Control		Treated	
	Mean	SEM	Mean	SEM	Mean	SEM	Mean	SEM
Albumin (ng/mL)	34.68	2.16	34.97	1.09	27.20	1.20	32.18	1.66*
TNF-α (ng/mL)	80.13	2.97	77.11	2.64	76.98	0.76	77.10	2.14
IgA (μg/mL)	72.8	5.74	73.71	6.26	67.45	5.24	74.26	6.48
GLU (mmol/L)	3.83	0.26	3.87	0.25	3.60	0.23	3.60	0.23
TG (mmol/L)	1.29	0.35	1.34	0.25	0.62	0.07	0.59	0.07
TC (mmol/L)	5.78	0.60	6.75	0.45	2.07	0.17	2.87	0.32*
AKP (King unit)	42.47	1.67	43.83	1.38	33.67	1.69	32.48	1.63
BUN (mmol/L)	3.71	0.41	3.88	0.33	4.44	0.28	4.47	0.35
GOT (King unit)	41.13	4.00	40.69	4.31	42.03	4.21	41.41	3.23
GPT (King unit)	17.02	1.53	15.96	2.62	14.36	1.89	13.71	1.60
MDA (mmol/L)	8.54	1.46	8.30	1.28	5.67	0.47	4.41	0.76
SOD (U/mL)	97.92	8.43	94.02	7.69	117.40	3.30	131.99	2.81*
Cortisol (ng/mL)	99.83	3.61	96.07	4.03	100.41	3.82	101.39	2.48
Endotoxin	0.21	0.05	0.21	0.03	0.22	0.07	0.21	0.06

*Values are statistically different (*P* < 0.05) compared with those of the control (feed with basal diet) piglets

*SOD*, superoxide dismutase; *MDA*, malondialdehyde; *GLU*, blood glucose; *TG*, triglyceride; *TC*, total cholesterol; *BUN*, blood urea nitrogen; *AKP*, alkaline phosphatase; *GOT*, aspartate aminotransferase; *GPT*, alanine aminotransferase; *TNF-α*, tumor necrosis factor α

### PD improved the gut morphology and intestinal barrier function in early weaned piglets

**Fig. 2:**
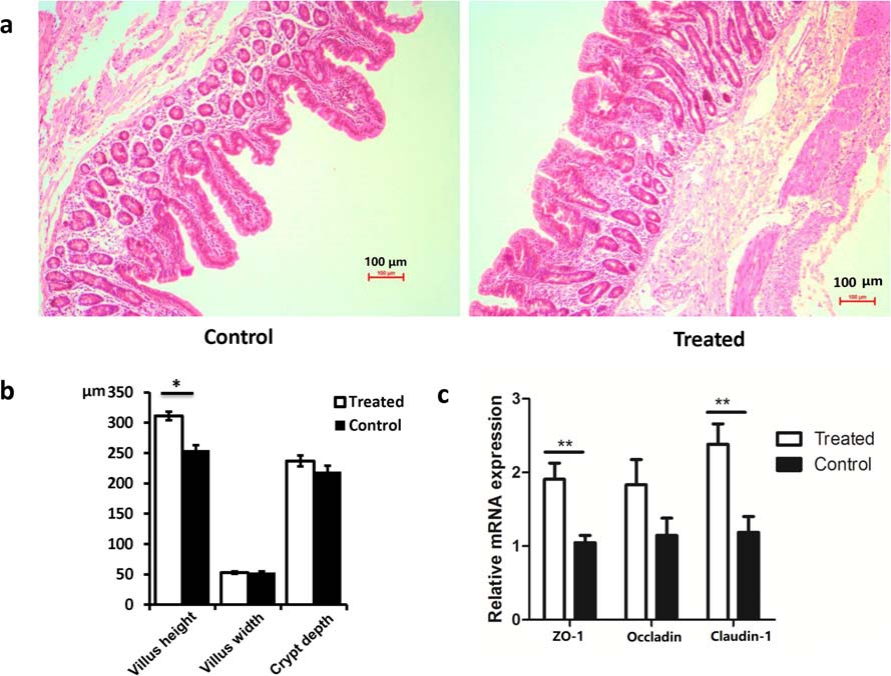
Representative image of HE staining analysis of the ileum tissues in weaned piglets at 42 days of age (**a**); statistical analysis of ileum tissues (**b**); and gene expression analysis of tight junction–associated proteins by qRT-PCR (**c**). Treated, feed by basal diet with 0.5% PD supplement; Control, feed with basal diet

At 42 days of age, no significant differences were observed in the villus width and crypt depth between the two groups. However, dietary PD supplementation significantly increased the villus height in early weaned piglets (*P* < 0.05; Fig. 2a, b). In addition, dietary PD significantly increased the gene expression of the tight junction–associated proteins Zonula occludens-1 (*ZO-1*) and claudin-1 (*P* < 0.01, Fig. 2c).

### PD affected gut microbiota composition in early weaned piglets

**Fig. 3:**
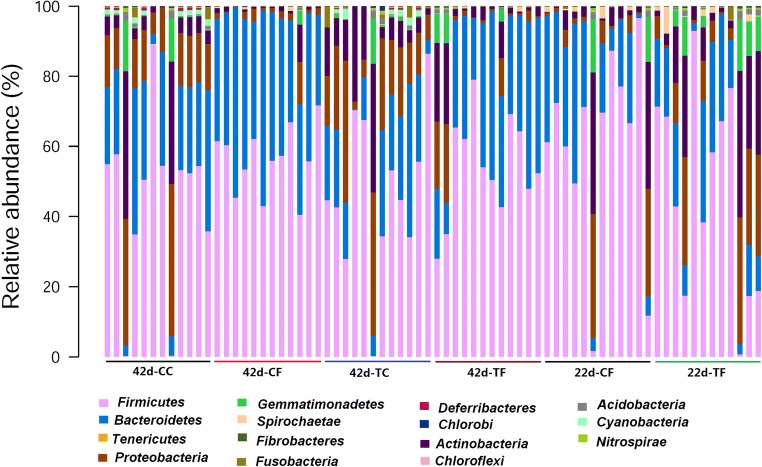
Distribution of gut microbiome composition for 25-day CF, 25-day TF, 42-day CC, 42-day TC, 42-day CF, and 42-day TF at the phylum level (each color represents one bacterial phylum). *CC*, ileum content collected from the control weaned piglets; *CF*, feces collected from the control weaned piglets; *TC*, ileum contents collected from the PD-treated piglets; *TF*, feces collected from the PD-treated piglets

**Fig. 4:**
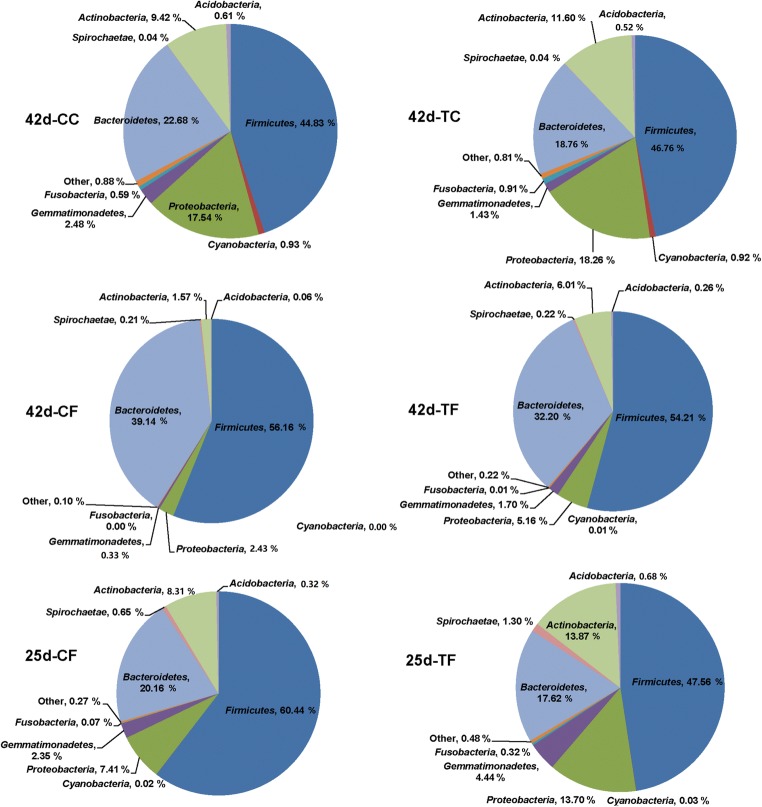
Mean phylum-level relative abundance, as detected by 16S rRNA sequencing (*n* = 12). *CC*, ileum content collected from the control weaned piglets; *CF*, feces collected from the control weaned piglets; *TC*, ileum contents collected from the PD-treated piglets; *TF*, feces collected from the PD-treated piglets

In this study, an average of 31,745 clean tags was obtained for each group, and the length of the sequences ranged between 425 and 429 bp (Supplemental Table S2). Furthermore, a mean of 521 observed species was obtained for each group, indicating adequate sequencing depth (Supplemental Table S2). At 25 days of age, the fecal microbiota appeared to be more diverse and had greater evenness in the PD-treated piglets than in the control weaned piglets, according to the Shannon and Simpson indices; however, the indices were not significantly changed (*P* > 0.05) (Supplemental Table S3). At 45 days of age, the Chao1 estimator, Good’s coverage, observed species, and Shannon index were not significantly changed (*P* > 0.05). *Bacteroidetes* and *Firmicutes* were the dominant phyla in samples from piglets in all groups (Fig. 3). At 25 days of age, the relative abundance of *Firmicutes* (47.56%) and *Bacteroidetes* (17.62%) was decreased, whereas that of *Proteobacteria* (13.70%), *Actinobacteria* (13.87%), *Spirochaetae* (1.30%), *Acidobacteria* (0.68%), and *Gemmatimonadetes* (4.44%) was increased in the PD-treated piglets. At 42 days of age, PD treatment tended to increase the abundance of *Firmicutes* but decreased the relative abundance of *Bacteroidetes* in the ileum contents in weaned piglets. Both *Bacteroidetes* and *Firmicutes* increased in the feces of piglets in response to PD treatment. Five phyla (*Proteobacteria*, *Actinobacteria*, *Spirochaetae*, *Acidobacteria*, and *Gemmatimonadetes*) increased in the feces of the PD-treated piglets (Fig. 4), but no significant differences were observed (Table 2). However, some phyla were present at low levels (< 0.05% abundance) and were classified into the category “other” (Fig. 4). In addition, PD significantly increased the relative abundance of *Cyanobacteria* (*P* = 0.024).

**Table 2:** Phylum-level taxonomic composition of the bacterial communities

Phylum	Feces at 25 days of age					Ileum content at 42 days of age						Feces at 42 days of age			
	25-day CF (%)		25-day TF (%)		*P* value	42-day CC (%)		42-day TC (%)		*P* value	42-day CF (%)		42-day TF (%)		*P* value
	Means	SEM	Means	SEM		Means	SEM	Means	SEM		Means	SEM	Means	SEM	
*Acidobacteria*	0.320	0.213	0.680	0.213	0.289	0.605	0.264	0.518	0.238	0.807	0.063	0.063	0.257	0.137	0.211
*Actinobacteria*	8.310	4.068	13.870	4.068	0.346	9.419	3.959	11.596	3.059	0.668	1.574	0.832	6.011	2.391	0.093
*Bacteroidetes*	20.160	3.987	17.620	3.987	0.623	22.681	3.769	18.764	3.496	0.454	39.137	2.654	32.197	3.560	0.132
*Chlorobi*	0.040	0.030	0.010	0.030	0.249	0.065	0.030	0.110	0.074	0.572	0.009	0.009	0.038	0.020	0.197
*Chloroflexi*	0.010	0.006	0.023	0.006	0.244	0.083	0.022	0.082	0.029	0.975	0.001	0.001	0.002	0.001	0.523
*Cyanobacteria*	0.020	0.010	0.027	0.010	0.540	0.934	0.203	0.923	0.301	0.976	0.001	0.001	0.013	0.005	0.024
*Fibrobacteres*	0.020	0.010	0.038	0.010	0.231	0.024	0.012	0.011	0.004	0.318	0.009	0.005	0.020	0.010	0.355
*Firmicutes*	60.440	8.090	47.558	8.090	0.282	44.825	7.139	46.759	6.488	0.843	56.161	2.730	54.211	4.244	0.703
*Gemmatimonadetes*	2.350	1.586	4.440	1.586	0.360	2.485	1.478	1.427	1.044	0.565	0.325	0.325	1.695	0.940	0.182
*Proteobacteria*	7.410	3.511	13.703	3.511	0.239	17.543	3.097	18.258	3.644	0.882	2.430	0.934	5.163	2.294	0.282

*CC*, ileum content collected from the control weaned piglets; *CF*, feces collected from the control weaned piglets; *TC*, ileum contents collected from the PD-treated piglets; *TF*, feces collected from the PD-treated piglets

The genera that were significantly affected by PD are shown in Table 3. At 25 days of age, the relative abundance of *Anaerotruncus* was significantly lower (*P* = 0.055), whereas that of *Lautropia* was significantly higher (*P* = 0.051) in the feces of the PD-treated piglets. At 42 days of age, the genera *C1_B04*5, *Defluviicoccus*, *Gardnerella*, and *Ruminococcaceae*_UCG_00*9* (0.007%, 0.018%, 0.003%, and 0.042%, respectively) were at least twofold higher than those in the ileum contents of the PD-treated piglets (0.000%, 0.001%, 0.000%, and 0.013%, respectively), and we did not detect *C1_B04*5 and *Gardnerella* in the ileum contents of the PD-treated piglets. The relative abundance of *Psychrobacter* was clearly higher (*P* = 0.036) than that in the ileum contents of the control weaned piglets. Fecal microbiome community analysis indicated that PD significantly increased the relative abundance of *Ambiguous*_taxa and *Collinsella* (*P* < 0.05) and decreased the abundance of *Prevotellaceae*_NK3B31_group, *Oscillospira*, and *Coprococcus*_1 (*P* < 0.05); meanwhile, *Prevotella*_7 (*P* = 0.061), *Mitsuokella* (*P* = 0.060), and *Lautropia* (*P* = 0.069) tended to increase in the PD-treated group (Table 3).

**Table 3:** Genus-level taxonomic composition of the bacterial communities

Genus	Feces at 25 days of age					Ileum content at 42 days of age						Feces at 42 days of age			
	25-day, CF (%)		25-day, TF (%)		*P* value	42-day CC (%)		42-day TC (%)		*P* value	42-day CF (%)		42-day TF (%)		*P* value
	Means	SEM	Means	SEM		Means	SEM	Means	SEM		Means	SEM	Means	SEM	
*Ambiguous_taxa*	3.006	0.887	3.046	0.571	0.970	1.299	0.348	1.198	0.314	0.833	1.318	0.133	2.136	0.227	0.005
*Anaerotruncus*	0.562	0.132	0.267	0.062	0.055	0.267	0.066	0.225	0.057	0.632	0.570	0.045	0.626	0.085	0.568
*C1_B045*	ND	ND	ND	ND	ND	0.007	0.003	0.000	0.000	0.032	ND	ND	ND	ND	ND
*Collinsella*	0.810	0.290	1.016	0.283	0.616	0.013	0.007	0.023	0.008	0.392	0.149	0.057	0.767	0.19	0.005
*Coprococcus_*1	0.205	0.122	0.140	0.062	0.640	0.137	0.027	0.074	0.020	0.080	0.396	0.050	0.226	0.05	0.024
*Defluviicoccus*	0.000	0.000	0.004	0.002	0.099	0.018	0.007	0.003	0.001	0.037	0.000	0.000	0.001	0.001	0.160
*Gardnerella*	ND	ND	ND	ND	ND	0.003	0.001	0.000	0.000	0.043	ND	ND	ND	ND	ND
*Lautropia*	0.012	0.008	0.013	0.006	0.912	0.011	0.007	0.012	0.006	0.915	0.000	0.000	0.003	0.002	0.069
*Longispora*	0.003	0.002	0.013	0.004	0.051	0.020	0.006	0.010	0.004	0.194	0.000	0.000	0.002	0.001	0.112
*Mitsuokella*	0.903	0.775	0.078	0.041	0.299	0.003	0.002	0.000	0.000	0.329	0.004	0.003	0.047	0.021	0.060
*Oscillospira*	0.037	0.012	0.054	0.016	0.394	0.073	0.027	0.054	0.014	0.532	0.566	0.100	0.298	0.062	0.033
*Prevotella_*7	0.771	0.466	0.496	0.191	0.590	0.342	0.074	0.595	0.292	0.411	0.443	0.182	1.646	0.581	0.061
*Prevotellaceae_*N*K3B31*_group	2.671	1.006	2.110	0.755	0.660	0.189	0.139	0.261	0.131	0.710	12.536	2.547	4.302	0.947	0.006
*Psychrobacter*	ND	ND	ND	ND	ND	0.010	0.003	0.298	0.129	0.036	ND	ND	ND	ND	ND
*Ruminococcaceae*_UCG_009	0.035	0.016	0.013	0.003	0.178	0.042	0.011	0.013	0.005	0.023	0.065	0.015	0.074	0.017	0.709

*ND*, not detected; *CC*, ileum content collected from the control weaned piglets; *CF*, feces collected from the control weaned piglets; *TC*, ileum contents collected from the PD-treated piglets; *TF*, feces collected from the PD-treated piglets

### PD caused functional changes in the gut microbiota

**Fig. 5:**
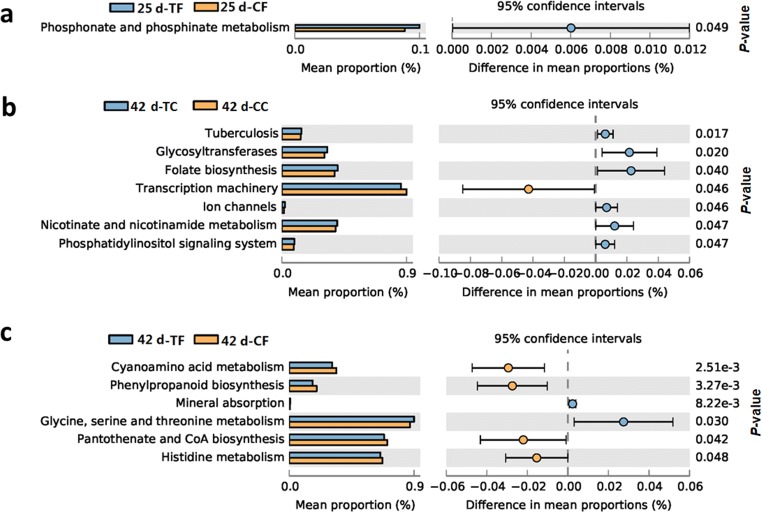
Significant KEGG category differences in the intestinal microbiota between the PD-treated piglets and the control animals, as conducted with the STAMP program. 25-day CF vs 25-day TF (**a**), 42-day CC vs 42-day TC (**b**), 42-day CF vs 42-day TF (**c**). *CC*, ileum contents collected from control weaned piglets; *CF*, feces collected from control weaned piglets; *TC*, ileum contents collected from the PD treated piglets; *TF*, feces collected from the PD-treated piglets. Bars on the left represent the proportion of each category, and category differences with a *P* value < 0.05 were considered to be significant

Within the KEGG categories, the phosphonate and phosphinate metabolism pathway was found to be significantly more abundant in the PD-treated piglets than the control weaned piglets at 25 days of age (Fig. 5a). At 42 days of age, ileum microbiota were enriched in six KEGG pathways (tuberculosis, glycosytransferases, folate biosynthesis, ion channels, nicotinate and nicotinamide metabolism, and phosphatidylinositol signaling system) in the PD-treated piglets compared with the control animals, whereas the transcription machinery pathway was more abundant in the control weaned piglets (Fig. 5b). Additionally, some KEGG pathways were abundant in the fecal microbiota of the PD-treated piglets, such as mineral absorption and glycine, serine, and threonine metabolism. However, the pathways of cyanoamino acid metabolism, phenylpropanoid biosynthesis, pantothenate and CoA biosynthesis, and histidine metabolism were more abundant in the fecal microbiota of the control weaned piglets (Fig. 5c).

### PD changed the gut SCFA composition in early weaned piglets

In accordance with the observed alterations in the fecal microbiota composition, differences in the levels of SCFAs between the PD-treated and control weaned piglets were also observed (Table 4). Acetic acid was a major SCFA in all the samples and was followed by propionic acid, isobutyric acid, isovaleric acid, and valeric acid. At 42 days of age, we found a significantly (*P* < 0.05) lower concentration of total fecal SCFAs for the control weaned piglets than for the PD-treated piglets. PD resulted in significantly (*P* < 0.05) higher concentrations of acetic acid and valeric acid in the feces of weaned piglets than of the control animals. However, no significant differences were observed in the ileum contents of piglets at 42 days of age as well as the feces of piglets at 25 days of age (Table 4).

**Table 4:** SCFA changes in early weaned piglets

Sample	Group	Acetic acid (mg/g)		Propionic acid (mg/g)		Butyric acid (mg/g)		Isobutyric acid (mg/g)		Valeric acid (mg/g)		Isovaleric acid (mg/g)		Total acid (mg/g)	
		Means	SEM	Means	SEM	Means	SEM	Means	SEM	Means	SEM	Means	SEM	Means	SEM
25-day feces	Treated	4.519	0.328	1.691	0.192	0.08	0.019	0.711	0.084	0.326	0.029	0.406	0.036	7.733	0.505
	Control	4.116	0.242	1.588	0.100	0.067	0.016	0.666	0.057	0.308	0.032	0.377	0.023	7.121	0.244
	*P* value	0.334		0.640		0.610		0.6570		0.685		0.503		0.292	
42-day ileum content	Treated	0.909	0.076	0.114	0.037	0.094	0.013	ND		0.165	0.010	0.150	0.024	1.431	0.096
	Control	1.132	0.143	0.089	0.007	0.070	0.012	ND		0.262	0.074	0.172	0.016	1.726	0.178
	*P* value	0.186		0.526		0.192		ND		0.216		0.445		0.16	
42-day feces	Treated	5.085	0.166	1.181	0.109	0.072	0.011	0.563	0.055	0.343	0.020	0.376	0.027	7.620	0.277
	Control	4.406	0.214	1.037	0.062	0.064	0.014	0.599	0.037	0.279	0.018	0.337	0.028	6.722	0.211
	*P* value	0.020		0.266		0.652		0.589		0.023		0.334		0.017	

*ND*, not detected; *SCFA*, short-chain fatty acid

### Correlation analysis between metabolic profiles and microorganisms

**Fig. 6:**
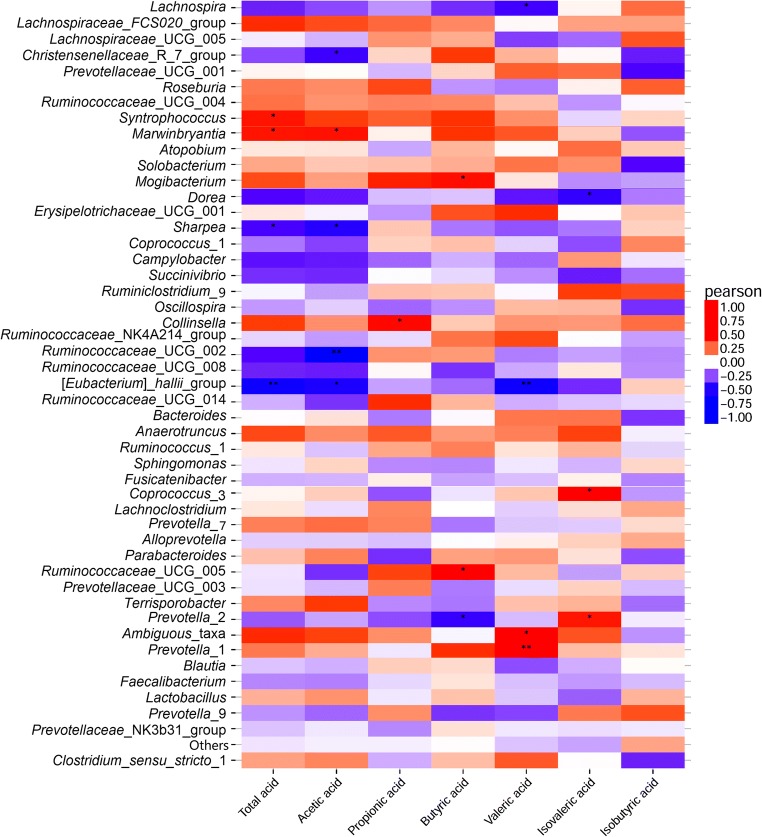
Correlation coefficients between the relative abundance of fecal bacteria genera (> 0.1% relative abundance) and SCFAs in piglets at 42 days of age. *P < 0.05, **P < 0.01

Correlation analysis (Fig. 6) showed a significant correlation between the piglet metabolic profile and the relative abundance of bacteria in the feces of piglets at 42 days of age. The relative abundance of *Prevotella*_1, *Ambiguous*_taxa, *Prevotella*_2, *Ruminococcaceae UCG*-005, *Coprococcus*_3, *Syntrophococcus*, *Marvinbryantia*, *Collinsella*, *Fusicatenibacter*, and *Mogibacterium* was positively correlated with the concentrations of SCFAs in the feces of piglets at 42 days of age, whereas *Prevotella*_2 was negatively correlated with the levels of butyric acid. In contrast, the relative abundance of *Ruminococcaceae* UCG-002, *Ruminococcaceae* UCG-008, *[Eubacterium]_hallii*_group, *Sharpea*, *Dorea*, and *Lachnospira* was negatively correlated with the concentrations of SCFAs. The relative abundance of *Prevotella*_1 and *Ambiguous*_taxa was positively correlated with the levels of butyric acid and that of *Marvinbryantia* was positively correlated with the levels of acetic acid.

## Discussion

In our previous work, we found that preventive supplementation with PD decreased the occurrence of diarrhea in early weaned piglets (Supplemental Table S4 and Supplemental Fig. S1). In the present study, we focused on changes in growth performance, serum biochemical parameters, and gut microbiota in post-weaning piglets after PD supplementation. The results confirmed that dietary supplementation with PD effectively alleviated weaning stress in piglets and improved the antioxidant capacity in the serum and the concentrations of organic acids in the intestinal contents.

As previously mentioned, PD is a resistant starch produced by heat treatment and is alpha-amylase-resistant, thus implying that it behaves like dietary fiber, producing SCFAs via fermentation in the large intestine and improving the intestinal environment through the microbiota. Thus, we expected that PD might favorably alter physiological functions in weaning piglets, thus resulting in decreased weaning stress, including increased feed efficiency and reduced diarrhea.

In pig production, feed cost remains a major production cost (60–70%) worldwide. Therefore, strategies used to improve feed efficiency not only increase the economic profitability of pig production but also decrease environmental pollution. The FCR corresponding to the ratio of inputs (feed intake) to outputs (body weight gain) is generally expressed as its inverse trait during growth and is widely used to measure feed efficiency in pig production (Broeke et al. 2019). In this study, dietary PD did not significantly affect the growth performance of early weaned piglets during the experimental period. Notably, dietary supplementation with PD significantly decreased the FCR of piglets from 28 to 42 days of age. The results are consistent with those of studies reported by Molist et al. (2014) and Zhao et al. (2018), which showed that moderate levels of dietary fiber are beneficial for the growth performance of pigs in the first 2 weeks after weaning (Molist et al. 2014; Zhao et al. 2018). Our results indicate that dietary supplementation with PD might improve the feed efficiency, although PD was added to the diet for only 28 days. Further research is needed to investigate the effects of long-term PD addition on FCR by examining the growth-finishing phase of pigs.

Evidence has demonstrated that early weaning caused significant deterioration of the intestinal morphology including villous atrophy and crypt hyperplasia, which are associated with reduced nutrition absorption and poor performance (Pluske and Williams et al. 1997). The small intestine is the major site of nutrient digestion and absorption in mammals. Villus height is used as an indirect indicator of intestinal epithelium cell function, because longer villi provide a larger absorptive area for nutrients. Moreover, tight junction proteins, such as occludin, claudin-1, and ZO-1, form tight junctions at the boundary of two adjacent cells to maintain intestinal integrity (Li et al. 2012). Weaning stress also causes severe decreases in the mRNA expression of *occludin*, *claudin-1*, and *ZO-1* in post-weaning pigs (Hu et al. 2013). In our previous study, when comparing to the suckling piglets, obvious decrease in villus height and mRNA expression of tight junction proteins, as well as significant increase in the content of serum cortisol, were observed in the early weaned piglets that confirmed the existence of weaning stress in post-weaning piglets (Zhu et al. 2013). In this study, we observed increases in villus height and the mRNA expression of tight junction genes (*claudin-1* and *ZO-1*) in ileum tissues when weaned piglets were fed dietary PD for 28 days, thus demonstrating that PD can be used to improve the intestinal morphology of early weaned piglets, and PD supplementation may help support intestinal functions such as nutrient digestion and absorption. Serum antioxidant capacity often represents the host’s response to endogenous oxidative damage. Improving antioxidant capacity is beneficial for relieving weaning stress (Wang et al. 2018). In this study, PD increased the concentration of SOD and tended to decrease the level of MDA in serum, thus indicating that PD can alleviate oxidative stress in weaned piglets.

Mammalian gastrointestinal tracts harbor widely diverse and active microbial communities that play important roles in nutrient digestion, vitamin production, maintenance of normal intestinal functions, regulation of the immune responses, and protection from pathogenic bacteria (Buffie and Pamer 2013; Kamada et al. 2013). During the weaning period, gut microbiota dysbiosis, including a loss of microbial diversity; a decrease in *Lactobacillus*; and an increase in *Acetivibrio*, *Dialister*, *Oribacterium*, *Prevotella*, and *Proteobacteriaceae*, including *E. coli*, have been reported by many studies (Konstantinov et al. 2006; Levesque et al. 2014; Mach et al. 2015). Dietary fiber can diversify the composition of the gut microbiome of weaning piglets (Chen et al. 2013; Niu et al. 2015; Zhao et al. 2018). For instance, in weaning piglets, diets in which expanded maize is replaced by 10% wheat bran fiber or 10% pea fiber increase the abundance of *Lactobacillus* in the ileum and that of *Bifidobacterium* in the colon (Chen et al. 2013), and a greater relative abundance of *Fibrobacteres* has been observed in the feces of weaned piglets fed a 5% wheat bran diet (Zhao et al. 2018). In addition, dietary corn bran supplementation increases the abundance of the phyla *Actinobacteria* and *Firmicutes*, but decreases that of *Bacteroidetes* in the feces of weaned piglets (Zhao et al. 2018). In this study, *Bacteroidetes* and *Firmicutes* were the two dominant phyla in piglets regardless of diet, in agreement with findings from a study by Niu et al. (2015). We observed that the relative abundance of the phyla *Firmicutes* and *Bacteroidetes* decreased, whereas that of other phyla including *Proteobacteria*, *Actinobacteria*, *Spirochaetae*, *Acidobacteria*, and *Gemmatimonadetes* increased in feces of the PD-treated piglets at 42 days of age. In addition, PD significantly increased the relative abundance of *Cyanobacteria*. The diverse results mentioned above may be related to different sources of fiber, as suggested by Zhao et al. (2018), who have reported that different fiber sources exert various effects on the gut health of swine depending on their physicochemical properties and chemical components. Notably, in our present study, at 25 days of age, the relative abundance of *Firmicutes* and *Bacteroidetes* decreased, whereas that of five phyla (*Proteobacteria*, *Actinobacteria*, *Spirochaetae*, *Acidobacteria*, and *Gemmatimonadetes*) increased in the feces of the PD-treated piglets, contrary to the results observed in the feces of the PD-treated piglets at 42 days of age. The differences might be associated with the term of PD feeding and the age of pigs, because bacterial abundance changes as pigs’ age, including an increase in the phyla *Firmicutes* and *Spirochaetes*, and a lower abundance of *Bacteroidetes*, *Actinobacteria*, and *Proteobacteria* (Kim et al. 2012).

At the genus level, the relative abundance of pathogenic organisms, including *Defluviicoccus* and *Gardnerella*, markedly decreased in the PD-treated group, whereas that of the commensal bacteria (genera *Psychrobacter* and *Prevotella*) usually observed in healthy gastrointestinal tracts increased in the ileum contents of piglets in response to PD treatment. Notably, these commensal bacteria have been shown to play important roles in nutrient absorption and immune response regulation. For instance, *Psychrobacter* spp. can be used as a probiotic to improve the feed efficiency, activity of digestive enzymes, and innate immune responses to *Epinephelus coioides* (Sun et al. 2011). Probiotic *Psychrobacter* spp. also increase microbial diversity in the gastrointestinal tract of *E. coioides* (Yang et al. 2011). In a recent study, *Psychrobacter namhaensis* SO89, a member of the genus *Psychrobacter*, has been suggested to be a new probiont in Nile tilapia feeds, because dietary *Psychrobacter namhaensis* SO89 not only improves growth performance, feed efficiency, and digestive enzyme activity but also upregulates the expression of immune-related genes (Makled et al. 2017). *Prevotella* species are dominant in the rumen and hindgut of cattle and sheep, where they facilitate the fermentation of protein and carbohydrate from foods. Consumption of dietary fiber improves glucose metabolism, which is associated with increased abundance of *Prevotella* (Kovatcheva-Datchary et al. 2015). Furthermore, the abundance of the genus *Collinsella* (phylum *Actinobacteria*) is positively correlated with circulating insulin, which affects host metabolism by altering cholesterol absorption, decreasing liver glycolysis, and increasing triglyceride synthesis (Clavel et al. 2014). Increased abundance of *Collinsella* has also been observed in overweight and obese pregnant women after low dietary fiber intake (Gomez-Arango et al. 2018). The variations in these genera, including higher levels of *Psychrobacter*, *Prevotella*, and *Collinsella*, may indicate that addition of PD can effectively inhibit the growth of harmful bacteria but promote the growth of commensal bacteria. Thus, increased abundance of commensal bacteria may also be associated with enhanced host metabolism in piglets in response to PD treatment, because enriched KEGG pathways such as mineral absorption and glycine, serine and threonine metabolism, and folate biosynthesis were observed in the PD-treated piglets compared with the control animals.

SCFAs, which are produced by gut bacteria from the fermentation of non-digestible carbohydrates, including dietary fiber, proteins, and peptides that are not digested and absorbed in the small intestine, have been shown to play pivotal roles in various aspects of host physiology. Acetic acid can resist pathogen invasion (such as *Pseudomonas aeruginosa* and *Listeria monocytogenes*) (Gonzalez-Fandos and Herrera 2014; Madhusudhan 2016; Nagoba et al. 2013), and valeric acid derivatives added in feed have been found to improve broiler performance, decrease feed conversion, and reduce the incidence of necrotic enteritis (Onrust et al. 2018). Therefore, we inferred that increased acetic acid and valeric acid in the feces of the PD group might have decreased the risk of pathogen infection, and these SCFAs might also have contributed to the improvement in feed efficiency in the weaning piglets. Our results are consistent with those from studies reported by Zhao et al. (2018) showing an increase in the levels of butyrate and valeric acid in feces in 5% wheat bran or corn bran fed piglets. As probiotics to stimulate SCFA production or improve tight junction integrity, dietary fiber has been used at the level of 5–10% (Chen et al. 2013; Mohamed et al. 2018). Similarly, in the present study, only 0.5% of PD was added and successfully improved TJs (tight junctions) and villi height.

Notably, increases in SCFA production are associated with enhanced growth of their bacteria producers. Members of genera including *Bacteroidales*, *Prevotella*, *Bifidobacterium*, *Akkermansia*, *Clostridium*, and *Streptococcus* appear to be responsible for the production of acetate (Koh et al. 2016; Louis et al. 2014). Fermentation of resistant maltodextrin is thought to contribute to butyrate production and increase *Bacteroidales* (Miyazato et al. 2016) and *Bifidobacterium* (Fastinger et al. 2008) in mice. In the present study, correlation analysis showed that the relative abundance of *Prevotella*_1, *Ambiguous*_taxa, *Prevotella*_2, *Ruminococcaceae UCG*-005, *Coprococcus*_3, *Syntrophococcus*, *Marvinbryantia*, *Collinsella*, *Fusicatenibacter*, and *Mogibacterium* was positively correlated with the SCFA concentrations in feces, thus suggesting that these bacteria have positive effects on weaned piglets’ health. Dietary supplementation with PD increased the relative abundance of SCFA-producing bacteria, indicating a shift toward a more adult pig-like intestinal environment associated with increased functional ability for carbohydrate degradation. Thus, the improvement in SCFAs in the feces of the PD-treated piglets might have resulted from changes in the colonization of gastrointestinal microflora.

In conclusion, dietary supplementation with PD improved the feed efficiency, elevated antioxidant capacity and promoted the development of intestinal morphology in early weaned piglets. Furthermore, PD significantly modulated the gut microbiotal composition and increased the production of SCFAs (especially acetic acid and valeric acid) in the feces of weaned piglets. In addition, correlation analysis revealed that the improvement in SCFAs was positively correlated with an increase in SCFA-producing bacteria. These findings demonstrated that PD improves the host intestinal homeostasis by modulating the gut microbiota composition, thus indicating that PD can be used to alleviate weaning stress in piglets.

## Electronic supplementary material

ESM 1 (PDF 505 kb)
