# Supplementary material for: Pyrodextrin enhances intestinal function through changing the intestinal microbiota composition and metabolism in early weaned piglets
Source: Appl Microbiol Biotechnol. 2020 Mar 3;104(9):4141–54. doi: 10.1007/s00253-020-10419-z (PMC7162831; doi:10.1007/s00253-020-10419-z)
Supplement: Supplementary file 1 — (PDF 505 kb) [file 253_2020_10419_MOESM1_ESM.pdf]

Journal name: Applied Microbiology and Biotechnology

Manuscript title: Pyrodextrin enhances intestinal function through changing the intestinal microbiota composition and metabolism in early weaned piglets

Lihui Zhu[1#], Rongrong Liao[1#], Weilong Tu[1,2#], Yonghong Lu[1*], Xuan Cai[1,2*]

[1]Institute of Animal Husbandry & Veterinary Science, Shanghai Academy of Agricultural Sciences, Shanghai 201106, PR China;
[2]Shanghai Engineering Research Center of Breeding Pig, Shanghai 201106, PR China

#These authors contributed equally to this work

*** Correspondence:**

Xuan Cai

E-mail:caixuan1985911@163.com, ORCID: 0000-0003-4413-8637,
Tel.: 86-21-62207862

*** Co-Correspondence:**

Yonghong Lu

E-mail:luyonghong@163.com, Tel.: 86-21-62207862

Shanghai Academy of Agricultural Sciences, 2901 Beidi Road, 201106, Shanghai, China.

Table S1 Primers for quantitative RT-PCR

Gene name	Primer (5'→3')	GenBank no.	Product (bp)
β-actin	F: TGCGGGACATCAAGGAGAAG	NM_001164650	216
	R: AGTTGAAGGTGGTCTCGTGG		
Occludin	F: GCTGGAGGAAGACTGGAT	NM_001163647	244
	R: ATCCGCAGATCCCTTAAC		
ZO-1	F: ACCCACCAAACCCACCAA	CK_453343	123
	R: CCATCTCTTGCCAAACTATC		
Claudin-1	F: AGAAGATGCGGATGGCTGTC	NM_001244539	193
	R: CCCAGAAGGCAGGAGAAGC		

Table S2 Alpha diversity indices of the bacterial communities

Group	Valid length of sequences (bp)	Clean tags	Valid percent (%)	OUT counts	Chao1 estimator	Good's coverage	Observed species	Shannon index	Simpson index
**25-d CF**	427	28,669	62.87	494	499	0.98	371	5.73	0.90
**25-d TF**	426	31,990	66.50	697	683	0.97	512	6.63	0.96
**42-d CI**	429	34,425	62.89	1,173	1,086	0.95	760	7.50	0.98
**42-d TI**	429	29,573	65.13	1,018	937	0.96	655	7.07	0.97
**42-d CF**	426	32,273	62.27	516	509	0.98	382	6.33	0.96
**42-d TF**	425	33,537	60.73	628	605	0.98	448	6.43	0.96
**Mean**	427	31,745	63.00	754	720	0.97	521	6.62	0.96

Table S3 Alpha diversity measures of bacterial communities

Item	Feces at 25 days of age			Ileum content at 42 days of age			Feces at 42 days of age		
	**25-d CF**	**25-d TF**	***P* value**	**42-d CI**	**42-d TI**	***P* value**	**42-d CF**	**42-d TF**	***P* value**
**Chao1 estimator**	499±57	683±89	0.26	1086±160	937±187	0.36	509±48	605±67	0.56
**Good's coverage**	0.98±0.00	0.97±0.00	0.35	0.95±0.01	0.96±0.01	0.45	0.98±0.00	0.98±0	0.56
**Observed species**	371±50	512±70	0.18	760±93	655±116	0.32	382±32	448±53	0.54
**Shannon index**	5.73±0.50	6.63±0.42	0.06	7.5±0.27	7.07±0.35	0.37	6.33±0.09	6.43±0.2	0.83
**Simpson index**	0.9±0.05	0.96±0.02	0.06	0.98±0.00	0.97±0.01	0.81	0.96±0	0.96±0.01	0.93

Diarrhea index

A total of 24 litters of landrace piglets (8-12 piglets per litter) with similar birth weights were randomly divided into C (Control), L（Low concentration），M（Middle concentration），H（High concentration）group, 6 litters per group. The piglets were kept with the sow in conventional farrowing pens and suckled until 21 d of age. From 14 to 56 d of age, piglets in the control weaning group had ad libitum access to the basal diet, while the L, M, and H group piglets were fed the basal diet supplemented with 250 mg/kg, 500 mg/kg, and 1000 mg/kg of PD, respectively. At 21 d of age, all the piglets were weaned and moved from the farrowing pens to nursery pens without mixing any litters.

From 22 d to 56 d of age, the piglet diarrhea was recorded once a day at 8:30-10:30 in the morning. The diarrhea score of piglets was also estimated according to **Table S4**. Firstly, observed the condition of the feces in the enclosure, and if the feces in the enclosure are formed, it is recorded as no diarrhea; if there is no formation or watery feces in the enclosure, the anus of the piglet is observed head by head. Recorded the ear number and the severity of diarrhea. The Diarrhea index was calculated as following:

Diarrhea index = sum of diarrhea scores of each group of piglets during the test period / (experimental days × number of piglets per group)

Table S4 Standard for diarrhea condition assessment

Degree of diarrhea	Fecal appearance	Diarrhea score
Normal	Dry, strip or granule	0
Mild	Soft manure, can be formed	1
Moderate	Thick, not formed, no separation of manure	2
Serious	Liquid, no shape, separation of manure, mucus or thick	3

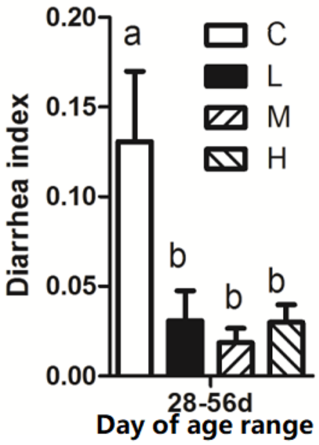

Fig.S1 Diarrhoea index of weaned pigs fed PD diets formulated with different concentrations.
C: Control, 0 mg/kg PD; L: Low concentration, 250 mg/kg PD; M: Middle concentration, 500 mg/kg PD; H: High concentration, 1000 mg/kg PD. [ab]Mean values not sharing the same superscript letters are significantly different from each other ($P \leq 0.05$).
